# Effects of subthreshold nanosecond laser therapy in age-related macular degeneration using artificial intelligence (STAR-AI Study)

**DOI:** 10.1371/journal.pone.0250609

**Published:** 2021-04-29

**Authors:** Verina Hanna, Jonathan Oakley, Daniel Russakoff, Netan Choudhry

**Affiliations:** 1 Faculty of Medicine, University of Toronto, Toronto, Ontario, Canada; 2 Vitreous Retina Macula Specialists of Toronto, Etobicoke, Ontario, Canada; 3 Voxeleron LLC, Pleasanton, California, United States of America; 4 Department of Ophthalmology & Vision Sciences, University of Toronto, Toronto, Ontario, Canada; Bascom Palmer Eye Institute, UNITED STATES

## Abstract

**Purpose:**

To investigate changes in retinal thickness, drusen volume, and visual acuity following subthreshold nanosecond laser (SNL) treatment in patients with age-related macular degeneration (ARMD).

**Design:**

Retrospective chart review.

**Methods:**

Patients with intermediate ARMD treated with a single session of SNL (2RT®, Ellex R&D Pty Ltd, Adelaide, Australia) were included. Swept-source optical coherence tomography (OCT) imaging (Triton; Topcon Medical Systems, Tokyo, Japan) was performed within 6 months before and after SNL treatment. Retinal layers were segmented using the artificial intelligence-enabled Orion® software (Voxeleron LLC, San Francisco, USA). The macular region was analyzed according to the Early Treatment Diabetic Retinopathy Study map. Mean difference and standard deviation in baseline and post-treatment retinal layer thicknesses are reported.

**Results:**

37 eyes from 25 patients were included in this study (mean age 74.7±9.2 years). An average of 51±6 spots were applied around the macula of each study eye, with a mean spot power of 0.33±0.04mJ. Increases in total retinal thickness were observed within the outer temporal and inferior sectors (P<0.05). Within the annulus, there was an increase in thickness of the sub-retinal pigment epithelial (RPE) space [0.88±2.41μm, P = 0.03], defined between the RPE and Bruch’s membrane. An increase in thickness of 1.13±2.55μm (P = 0.01) was also noted in the inferior sector of the photoreceptor complex, defined from the inner and outer segment junction to the RPE. Decreases in thickness were observed within the superior sector of the inner nuclear layer (INL) [-1.08±2.55μm, P = 0.01], and within the annulus of the outer nuclear layer (ONL) [-1.44±3.55μm, P = 0.02].

**Conclusions:**

At 6 months post-SNL treatment, there were sectoral increases in OPL, photoreceptor complex, and sub-RPE space thicknesses and sectoral decreases in INL and ONL thicknesses. This pilot study demonstrates the utility of OCT combined with artificial intelligence-enabled software to track retinal changes that occur following SNL treatment in intermediate ARMD.

## I. Introduction

Age-Related Macular Degeneration (ARMD) affects between 7.4–30% of those aged 45 to 85, and is a common cause of blindness [1]. Early ARMD is characterized by a thickened Bruch’s membrane (BM), which results from a deterioration in its transport functions. This leads to the accumulation of lipid-rich extracellular material between the retinal pigment epithelium (RPE) and BM, which are seen on fundus examination as drusen.

Nutritional supplementation and lifestyle interventions (e.g. smoking cessation, physical activity) can slow the progression of ARMD in its early and intermediate stages [2,3]. In its advanced forms, this disease results in RPE atrophy (dry ARMD) or abnormal growth of choroidal vessels into BM (wet ARMD) [4].

Recently, subthreshold nanosecond laser (SNL) was investigated as a potential treatment for decreasing ARMD progression. A preliminary study demonstrated laser-induced regulation of extracellular matrix remodeling pathways, which resulted in thinning of BM [5,6]. In humans, *Jobling et al*. found a reduction in drusen area with no subsequent damage to photoreceptors following laser treatment. The Laser Intervention in Early Stages of Age-Related Macular Degeneration (LEAD) study was the first multi-centered randomized controlled trial to investigate the impact of SNL treatment in patients with intermediate ARMD. This study found that SNL treatment did not reduce the overall rate of progression to late ARMD. *Post hoc* subgroup analysis suggested significant treatment effect modification based on the presence of reticular pseudodrusen (RPD), where eyes without these subretinal drusenoid deposits exhibited a lower rate of progression to late ARMD [7]. Further study is required to definitively delineate this relationship, given the limitations of this retrospective analysis, including the loss of randomization [8,9].

Identification of RPD requires the use of at least two different imaging modalities, such as optical coherence tomography (OCT) and color fundus photography [10]. OCT is an invaluable tool for the assessment of ARMD-related retinal changes in vivo, and for identifying patients who may benefit from SNL treatment [7,10]. Central to detecting these changes is accurate segmentation of each anatomic layer, which can be laborious and time-consuming to complete manually. Artificial intelligence-enabled software has been developed and validated to improve the accuracy of automated segmentation of distinct retinal layers. When compared to manual segmentation of the macula, this software can accurately identify seven retinal layer interfaces [11].

This study presents a real-world evaluation of the structural and functional outcomes of SNL treatment, and uses artificial intelligence to maximize the utility and accuracy of OCT in tracking changes over time. The purpose of this investigation is to determine the effect of SNL treatment on retinal layer thickness, retinal volume, and visual acuity in patients with intermediate ARMD.

## II. Methods

### A. Study population

This pilot study is a single-center retrospective chart review of all patients who underwent SNL treatment and completed their 6-month post-treatment follow up visit. Ethics approval for this study was obtained from the CIRBI Institutional Review Board and research adhered to the tenets of the Declaration of Helsinki. Consent was not obtained from patients included in this retrospective chart review as all data were analyzed anonymously.

Patients were included in this study according to the following criteria: 1) age 50 to <95 years at the time of SNL treatment, 2) intermediate ARMD with at least one druse >125μm within 1500μm from the fovea, 3) pupil dilation of at least 5mm possible in treatment eye, 4) single session of SNL treatment, 5) OCT scans available within 6 months prior to SNL treatment, and 6) OCT scans available at 6 months post-treatment.

Patients were excluded according to the following criteria: 1) presence of RPD, 2) any evidence of advanced ARMD, 3) other macular disease with subretinal deposits not typical of ARMD, 4) ocular disease other than ARMD that significantly compromised the ability to treat or visualize the fundus, 5) baseline OCT scan taken >6 months prior to SNL treatment, and 6) OCT scans taken with a device other than the specified device.

### B. SNL treatment

A single session of SNL treatment was performed using the retinal rejuvenation therapy device (2RT®, Ellex R&D Pty Ltd, Adelaide, SA, Australia). This device uses a 532nm laser with 3-nanosecond pulse duration to induce a non-thermal injury confined to the RPE. The laser was applied along the arcades as previously described with a target of 50 spots per eye [7]. The power was titrated starting at 0.25mJ and increased in 0.05mJ increments until a faint white laser spot was visible, then the power was reduced by 0.05mJ for the treatment.

### C. Imaging

Swept-source OCT using a commercial OCT device (Triton; Topcon Medical Systems, Tokyo, Japan) was performed within 6 months prior to SNL treatment and at 6 months post-SNL treatment. The macular region was captured with a 7mm x 7mm (512x256) volume scan centered on the macula.

### D. OCT segmentation & image analysis

Following image acquisition, structural OCT layer segmentation was performed using Orion^TM^ (Voxeleron LLC, San Francisco, CA, USA). This artificial intelligence-enabled software performed automated segmentation of the retina into individual layers. Retinal layer thickness and volume in the central macular area were calculated by the software. The retina was segmented into the following seven layers: 1) retinal nerve fiber layer (RNFL), 2) ganglion cell and inner plexiform layer (GC-IPL), 3) inner nuclear layer (INL), 4) outer plexiform layer (OPL), 5) outer nuclear layer (ONL), 6) photoreceptors (PR), and 7) the sub-retinal pigment epithelium (RPE) space, defined between the RPE and Bruch’s membrane complex. Total retinal thickness was defined from the inner limiting membrane to BM. This software was previously validated in eyes with early and intermediate ARMD [12].

**Fig 1:**
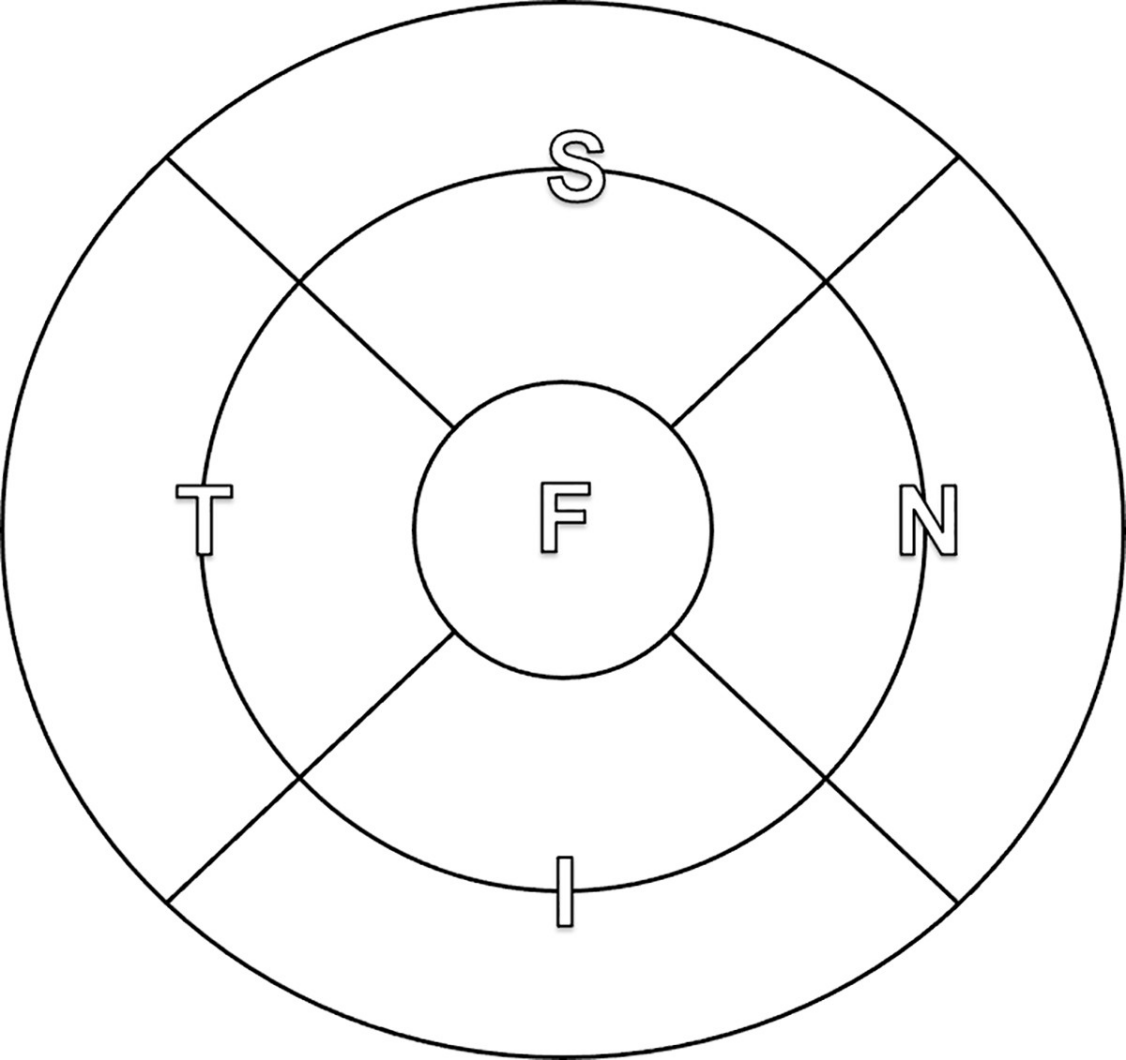
Analysis of the macular region according to the ETDRS map, centered on the fovea (F). The annular region is divided into a 3mm-diameter inner annulus and a 6mm-diameter outer annulus. Each annulus is further divided into superior (S), nasal (N), inferior (I), and temporal (T) sectors.

**Fig 2:**
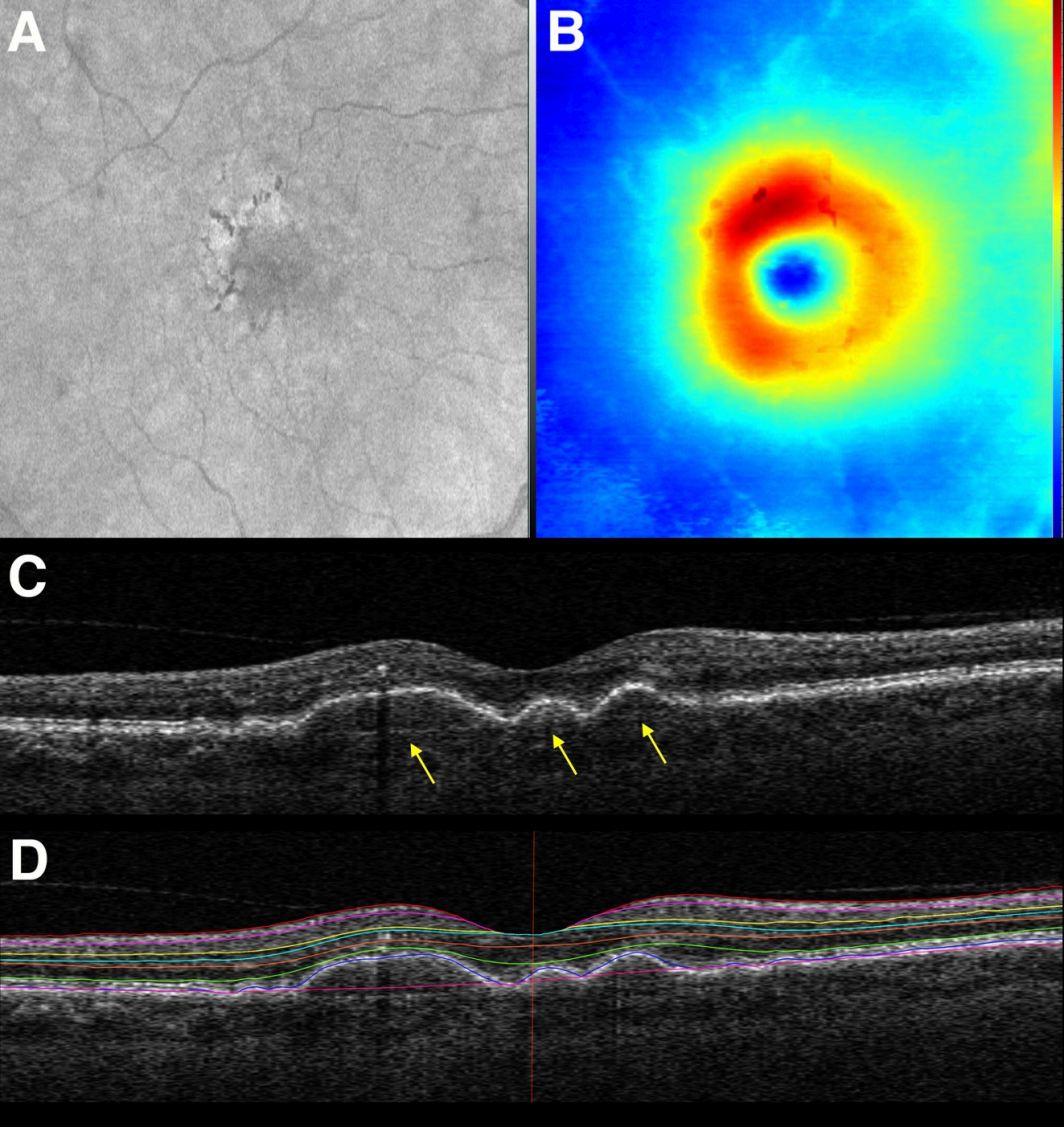
A) En face OCT of the macular region. B) Corresponding thickness map of ILM to RPE. Drusen are seen as areas of thinning peripheral to the fovea. C) OCT B-scan with drusen deposits seen below the RPE (arrows). D) OCT B-scan with artificial intelligence-enabled segmentation of retinal layers.

The software analyzed the retina according to the Early Treatment Diabetic Retinopathy Study (ETDRS) map, automatically centered on the fovea (Fig 1). Retinal thicknesses were reported within each sector of the ETDRS map, as well as combined sectors making up total, inner, and outer annuli. Average thicknesses can be converted to total volumes using their areas, a single multiplicative factor for a given zone. A sample of the software output can be seen in Fig 2.

### E. Statistical analysis

Descriptive statistics, including means, mean differences, and standard deviations, were applied for all variables. Paired two-tailed t-tests were used to compare baseline and post-treatment data in the peri-foveal annular region and within each sector of the ETDRS map. Subgroup analysis for all variables was performed for eyes demonstrating BCVA ≥20/40 at baseline, as per the inclusion criteria of the LEAD study [7]. A P-value <0.05 was considered statistically significant for all analyses.

## III. Results

**Fig 3:**
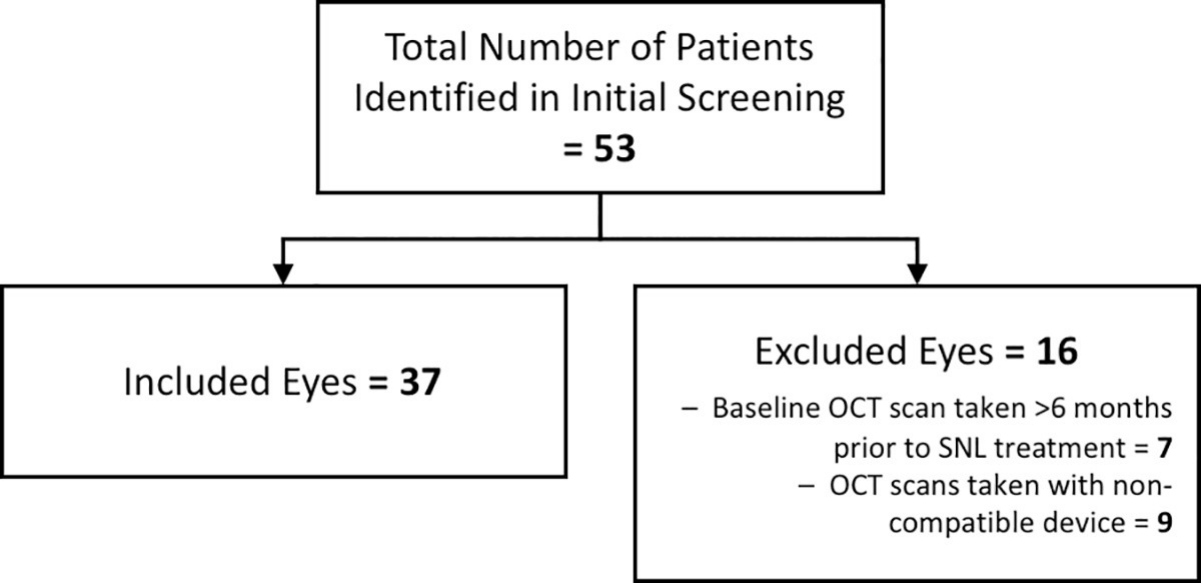
Flow diagram of study participants and reasons for exclusion.

A total of 37 eyes from 25 patients with intermediate ARMD were included in this retrospective chart review. Reasons for exclusion are listed in Fig 3. Participants were on average 74.7 ± 9.2 years of age at the time of SNL treatment. SD-OCT imaging was performed within 6 months (mean 35.4 ± 33.2 days) prior to SNL treatment and at 6 months (171.8 ± 33.9 days) post-treatment. Participant characteristics and SNL treatment details are found in Table 1.

**Table 1:** Characteristics of study participants and details of SNL treatment.

	Mean (SD) N (%)
**Age of Participants**	74.3 (9.33)
*Sex*	
**Male (%)**	8 (32%)
**Female (%)**	17 (68%)
**Baseline Visual Acuity (LogMAR)**	0.24 (0.25)
**Number of days between baseline OCT and SNL treatment**	35.4 (33.2)
**Number of days between SNL treatment and post-treatment OCT**	171.8 (33.9)
*SNL Treatment*	
**Number of Spots**	50.8 (6.0)
**Spot Power (mJ)**	0.33 (0.04)
**Average Total Energy (mJ)**	16.86 (2.95)

### A. Retinal thickness

**Fig 4:**
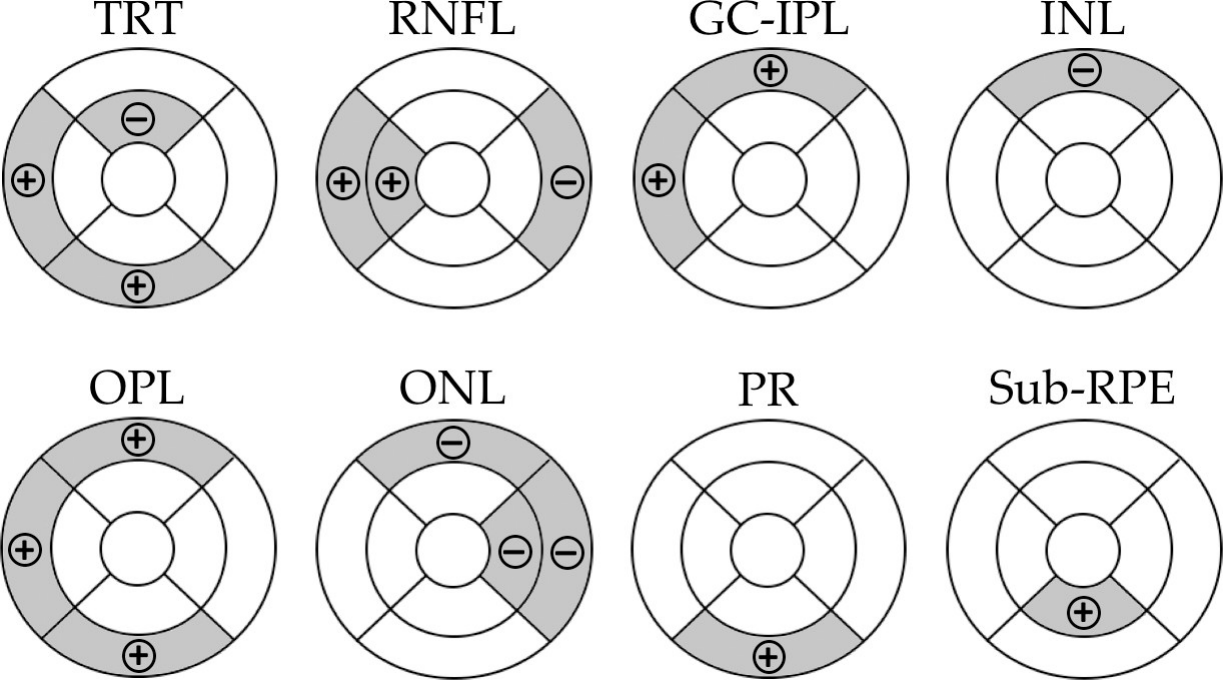
ETDRS maps demonstrating sectors that showed changes in retinal thickness following laser therapy. Shaded sectors indicate a statistically significant change in retinal thickness; (+) indicates an increase in thickness and (–) indicates a decrease in thickness.

Overall, increases in retinal thickness was observed in the GC-IPL, OPL, PR, and sub-RPE layers. Retinal thinning was seen in the INL and ONL layers. There were mixed changes in the RNFL, with both increases and decreases in thickness observed in different areas. Mean differences in retinal thickness from baseline to post-treatment follow-up are reported in Table 2. Within the peri-foveal region, there were statistically significant increases in thickness of the OPL (P = 0.02) and sub-RPE (P = 0.03) layers, while thinning was observed in the ONL (P = 0.02). Fig 4 details the sectoral changes in each layer according to the ETDRS map. These images show that the statistically significant changes seen in a quadrant correspond, and are further localized, to a particular sector of the ETDRS map. The total retinal thickness, which is a sum of the thickness of each retinal layer, exhibits areas of both increases and decreases in thickness. The thinning observed in the inner superior sector is due to the fact that the thinning of the INL and ONL was greater than the increase in thickness of the other layers.

**Table 2:** Mean difference ± standard deviation in thickness between baseline and post-treatment macular scans.

	Inferior	Nasal	Superior	Temporal	Annulus
**Total Retinal Thickness**	3.25 ± 10.6	-1.38 ± 10.2	-0.81 ± 9.36	2.33 ± 6.93*	0.85 ± 3.46
**RNFL**	-0.29 ± 4.39	-1.46 ± 5.30	-0.55 ± 4.16	2.02 ± 3.06*	-0.07 ± 2.46
**GC-IPL**	0.28 ± 3.77	-1.70 ± 5.30	1.79 ± 4.35*	1.73 ± 3.35*	0.53 ± 2.50
**INL**	-0.24 ± 3.27	-0.70 ± 2.24	-1.09 ± 2.55*	-0.80 ± 2.67	-0.71 ± 2.12
**OPL**	1.64 ± 4.19*	0.89 ± 5.13	1.83 ± 3.94*	0.79 ± 2.99	1.29 ± 3.11*
**ONL**	-0.93 ± 4.65	-1.56 ± 3.86*	-2.37 ± 5.60*	-0.88 ± 4.08	-1.44 ± 3.55*
**PR**	1.13 ± 2.55*	0.53 ± 3.59	0.22 ± 1.60	-0.39 ± 2.57	0.37 ± 1.59
**Sub-RPE**	1.66 ± 4.36*	2.62 ± 7.64*	-0.64 ± 3.46	-0.14 ± 3.74	0.88 ± 2.41*

* indicates a statistically significant result (P<0.05).

Subgroup analysis was performed for eyes demonstrating BCVA ≥20/40 at baseline (Table 3). An increase in total retinal thickness was seen in the inferior quadrant and within the inner nasal sector (3.42 ± 7.70, P = 0.02). Increase in OPL thickness was confined to the outer superior (1.47 ± 3.08, P = 0.02) and inferior (1.57 ± 3.78, P = 0.03) sectors. The sub-RPE space displayed increased thickness in the entire peri-foveal region, as well as in the inner inferior (4.84 ± 11.8, P = 0.04) and nasal (6.19 ± 13.1, P = 0.02) sectors. Increased thickness of the GC-IPL and mixed changes in the RNFL persisted following subgroup analysis. Contrary to the previous analysis, however, there were no areas of overall thinning in the ONL, and significant thinning was observed only in the outer superior sector of the INL (-0.89 ± 1.95, P = 0.02).

**Table 3:** Subgroup analysis of eyes with BCVA ≥ 20/40.

	Inferior	Nasal	Superior	Temporal	Annulus
**Total Retinal Thickness**	3.17 ± 8.00*	-0.39 ± 9.17	0.39 ± 5.48	1.83 ± 6.20	1.25 ± 3.40
**RNFL**	-0.07 ± 4.50	-1.69 ± 4.75	-0.37 ± 3.29	1.39 ± 2.74*	-0.18 ± 2.29
**GC-IPL**	0.89 ± 2.77	-1.14 ± 4.74	2.02 ± 3.28*	2.04 ± 3.53*	0.95 ± 185*
**INL**	-0.40 ± 2.89	-0.29 ± 1.60	-0.73 ± 2.27	-0.56 ± 2.47	-0.50 ± 1.78
**OPL**	1.32 ± 3.71	0.65 ± 4.82	1.26 ± 3.41	0.17 ± 2.88	0.85 ± 2.95
**ONL**	-1.08 ± 4.60	-1.01 ± 3.55	-1.64 ± 5.69	-0.59 ± 4.14	-1.08 ± 3.61
**PR**	1.24 ± 2.45*	0.08 ± 1.86	0.04 ± 1.41	-0.41 ± 2.70	0.24 ± 1.38
**Sub-RPE**	1.26 ± 3.88	3.01 ± 8.25	-0.19 ± 2.71	-0.20 ± 2.79	0.97 ± 2.52*

Mean difference ± standard deviation in thickness between baseline and post-treatment macular scans.

* indicates a statistically significant result (P<0.05).

### B. Retinal volume

Changes in retinal volume are reported in Table 4. Sub-RPE volume, where drusen is known to accumulate, was not significantly different within the inner (P = 0.15) or outer (P = 0.08) annulus following SNL treatment. Similarly, there was no significant change in total retinal volume within this study (P>0.05).

**Table 4:** Total retinal volume and sub-RPE volume before and after SNL treatment within a 3mm-diameter and 6mm-diameter annulus centered on the macula.

	Total Retinal Volume		Sub-RPE Volume	
	3mm circle	6mm circle	3mm circle	6mm circle
**Pre-Treatment**	2.095 ± 0.152	7.783 ± 0.442	0.113 ± 0.138	0.208 ± 0.207
**Post-Treatment**	2.081 ± 0.148	7.783 ± 0.463	0.124 ± 0.151	0.230 ± 0.218
**P-value**	0.06	1.00	0.15	0.08

* indicates a statistically significant change in retinal volume following laser therapy (P<0.05).

Subgroup analysis of retinal volumes in eyes with baseline BCVA ≥20/40 revealed a conservative, but significant, increase in volume within the inner annulus (mean difference 0.019 ± 0.033, P = 0.005) of the sub-RPE space. An increase in the outer annulus volume of the sub-RPE space was also observed (0.029 ± 0.072, P = 0.04). There was no significant change in overall total retinal volume, which included the foveal area in the calculation.

### C. Visual acuity

Post-treatment visual acuity (0.213 ± 0.214) did not significantly differ from baseline (0.240 ± 0.247, P = 0.24). This result was unchanged following subgroup analysis (P = 0.85).

## IV. Discussion

This study demonstrates that changes in the retina are detectable within 6 months post-SNL treatment in patients with intermediate ARMD. Sectoral increases in thickness were observed in the RNFL, as well as in the GC-IPL, OPL, PR, and sub-RPE layers. Sectoral thinning was observed in the INL and ONL. Further, sub-RPE volume was unchanged in this study, which implies no change in drusen volume. However, significant changes in retinal thickness and volumes were observed following exclusion of the central sub-field. Results of the subgroup analysis of patients with BCVA ≥20/40 showed fewer areas of retinal thinning in the INL and ONL, as well as an increase in sub-RPE volume within the peri-foveal region. This investigation highlights the utility of using artificial intelligence-enabled software to identify and track early structural changes on OCT imaging that occurred following SNL treatment.

Additionally, there was no detectable change in visual acuity as a result of SNL treatment within this study period. The lack of change in functional outcomes indicates that SNL treatment was not harmful during the course of this short-term investigation. However, further study is warranted to determine if this treatment results in improvement, or at least, ongoing stability of visual acuity.

The natural history of drusen and associated atrophy has been previously documented. *Wu et al*. investigated changes in retinal thickness prior to the development of drusen-associated atrophy in eyes with intermediate ARMD. This study described the features of nascent geographic atrophy, which include subsidence of the OPL and INL with or without the development of a wedge-shaped band within the limits of the OPL [13]. Additionally, other studies, including the LEAD trial, defined drusen-associated atrophy as thinning of the INL, OPL, ONL, and RPE [7,14,15]. In the present investigation, an increase in thickness was observed in the OPL and sub-RPE layers, suggesting a potential deviation from the natural history. Additional study is required to observe the long-term progression of ARMD, and the impact of SNL treatment.

Previous work has demonstrated errors in existing automated segmentation algorithms, necessitating the development of an effective and reproducible method for measuring retinal thickness [16]. This is of particular utility in longitudinal study, which requires analyzing several OCT scans for each patient. *Lamin et al*. have demonstrated that the accuracy of the Orion® software is sustained in the presence of macular pathology, such as ARMD, and changes in retinal layers are detectable in eyes with ARMD compared to control eyes [12]. As such, automated segmentation of OCT scans using artificial intelligence is an indispensable tool for efficiently and accurately completing this process.

The limitations of this investigation include the variation between patients in time elapsed between imaging and SNL treatment. While this is difficult to standardize due to the real-world nature of this study, a cut-off of 6 months between baseline imaging and SNL treatment was selected to avoid significant increases in drusen area that have been shown to occur over longer periods of time, as well as to evaluate any early responses to the treatment [17,18]. Since ARMD is known to be a slowly progressing disease, the impact of SNL treatment may not be captured at this time. As such, long-term observation is required to assess changes. Additionally, future study should investigate whether the patient’s smoking status may modify the outcomes of treatment.

Interpretation of automated segmentation results of very thin layers such as the OPL should be considered with caution. Small errors can lead to seemingly large changes. Additionally, when the OCT beam is not directly incident on this layer, structure such as Henle’s fibers are revealed making the layer appear thicker on one side of the fovea and thinner on the other. Small changes in pupil entry position of the OCT beam can cause this, and the resulting retinal image appears tilted.

While the RPE and BM were segmented accurately based on our review, the inner segment-outer segment junction was less visible with the pathology and the segmentation could look overly smooth. In all such cases, it may make more sense to analyze a retinal complex; for instance, analyzing the ONL and the PR complex as a single thickness, in much the same way the GC-IPL is used given that the interface of the ganglion cells and their dendrites in the IPL is barely visible using unaveraged OCT.

At 6 months post-SNL treatment, there were sectoral increases in retinal thickness in most layers, with the exception of the INL and ONL, where retinal thinning occurred. This study contributes to the evolving understanding of SNL treatment in ARMD, through the use of OCT imaging enhanced with artificial intelligence-enabled segmentation. This allows for longitudinal assessment of retinal changes that occur in response to SNL treatment in eyes with intermediate ARMD. Greater understanding of the changes that occur in the retina over time can contribute to the evidence for the effectiveness of SNL as a potential intervention in ARMD.

## Supporting information

S1 Dataset Data set used in the present investigation. (XLSX)
